# Acceptability and feasibility of a 12-week yoga vs. educational film program for the management of restless legs syndrome (RLS): study protocol for a randomized controlled trial

**DOI:** 10.1186/s13063-019-3217-7

**Published:** 2019-02-15

**Authors:** Terry Kit Selfe, Sijin Wen, Karen Sherman, Maryanna Klatt, Kim E. Innes

**Affiliations:** 1 Health Science Center Libraries, University of Florida, PO Box 100206, Gainesville, FL 32610 USA; 2 Department of Biostatistics, West Virginia University School of Public Health, HSC N, PO Box 9190, Morgantown, WV 26506 USA; 3 Kaiser Permanente Washington Health Research Institute, Seattle, WA USA; 4 Department of Family Medicine, The Ohio State University College of Medicine, Columbus, OH USA; 5 Department of Epidemiology, West Virginia University School of Public Health, HSC N, PO Box 9190, Morgantown, WV 26506 USA

**Keywords:** Restless legs syndrome, Yoga, Mind-body therapy, Behavioral intervention, Feasibility, Acceptability, Sleep, Mood, Quality of life

## Abstract

**Background:**

Restless legs syndrome (RLS) is a common and burdensome sleep disorder associated with profound impairment of health, well-being, and quality of life. Unfortunately, the medications used for RLS management carry risk of serious side effects, including augmentation of symptoms. Yoga, an ancient mind-body discipline designed to promote physical, emotional, and mental well-being, may offer a viable, low-risk new treatment. The primary objectives of this pilot, parallel-arm, randomized controlled trial (RCT) are to assess the acceptability and feasibility of a 12-week yoga vs. educational film program for the management of RLS.

**Methods:**

Forty-four adults with confirmed moderate to severe RLS will be recruited and randomized to a 12-week yoga (*n* = 22) or standardized educational film program (*N* = 22). Yoga group participants will attend two 75-min *Iyengar* yoga classes per week for the first 4 weeks, then one 75-min class per week for the remaining 8 weeks, and will complete a 30-min homework routine on non-class days. Educational film group participants will attend one 75-min class per week for 12 weeks and complete a daily RLS treatment log; classes will include information on: RLS management, including sleep hygiene practices; other sleep disorders; and complementary therapies likely to be of interest to those participating in a yoga and sleep education study.

Yoga and treatment logs will be collected weekly. Feasibility outcomes will include recruitment, enrollment, and randomization rates, retention, adherence, and program satisfaction. Program evaluation and yoga-dosing questionnaires will be collected at week 12; data on exploratory outcomes (e.g., RLS symptom severity (IRLS), sleep quality (PSQI), mood (POMS, PSS), and health-related quality of life (SF-36)) will be gathered at baseline and week 12.

**Discussion:**

This study will lay the essential groundwork for a planned larger RCT to determine the efficacy of a yoga program for reducing symptoms and associated burden of RLS. If the findings of the current trial and the subsequent larger RCTs are positive, this study will also help support a new approach to clinical treatment of this challenging disorder, help foster improved understanding of RLS etiology, and ultimately contribute to reducing the individual, societal, and economic burden associated with this condition.

**Trial registration:**

ClinicalTrials.gov, ID: NCT03570515. Retrospectively registered on 1 February 2017.

**Electronic supplementary material:**

The online version of this article (10.1186/s13063-019-3217-7) contains supplementary material, which is available to authorized users.

## Background

Restless legs syndrome (RLS) is a distressing sleep and sensorimotor disorder characterized by a compelling urge to move the legs, which is usually accompanied by unpleasant sensations in the legs that begins or worsens during periods of inactivity, is worse at night, and is at least partially relieved by movement [1]. As detailed in our recent reviews [2, 3], RLS affects up to 29% of US and European adults, with estimated prevalence rates averaging 12% in the general population, and 19.5% in primary care patients [3]. Up to 65% or more of those affected suffer moderate to severe symptoms [3]. While increasingly recognized as a disorder of significant clinical and economic import [3–7], the etiology of RLS remains poorly understood [8–10]. Currently, the primary underlying causes of RLS are thought to be genetic predisposition, dopaminergic dysfunction, and deficiencies in iron metabolism [8–14], although these factors have to date offered only a partial explanation [2]. As discussed in our recent papers [2, 3, 15], emerging evidence suggests that autonomic and hypothalamic-pituitary-adrenal (HPA) axis dysregulation may also play an important role in RLS pathogenesis and progression.

RLS is associated with significant sleep disturbance [6, 16, 17], the most common presenting complaint of those seeking medical care for RLS [18, 19], and is a major cause of chronic sleep loss [20]. Recent studies suggest that up to 40% of those with insomnia suffer from RLS [21, 22]. Likewise, mood disturbance is common in those affected by RLS [16, 23], and can both result from, and contribute to, sleep deficits [24]. Recent community-based studies suggest that RLS may also be linked, in a bidirectional manner, to cardiovascular disease (CVD), stroke, and key components of the metabolic syndrome [2, 8, 9, 25], associations that may be in part mediated by RLS’s adverse effects on sleep and mood [26, 27]. RLS leads to significant impairment of daily functioning and quality of life, increased health-related costs, and declines in productivity that are comparable to those reported in other serious chronic disorders, including diabetes, hypertension, Parkinson’s disease, and stroke [6, 7, 26, 28, 29]. Collectively, these studies suggest that RLS is a serious, chronic condition of major public health import, affecting a large percentage of the adult population and exacting a significant toll in terms of health, quality of life and economic cost.

There is no cure for RLS, with current treatments aimed at symptom management. Pharmaceuticals, notably dopaminergic agents and anti-seizure medications (α2δ ligands) remain first-line treatments for RLS, with opioids and, less commonly, benzodiazepines, used as second-line therapies [25, 26, 30–32]. Unfortunately, all medications used for RLS management carry risk of serious side effects (with 6–80% of patients affected, depending on the medication and duration of treatment) [26, 31, 33]. Among the most troubling is augmentation of symptoms, a serious clinical problem which has been reported for all dopaminergic drugs and certain opioids, with risk increasing with longer treatment duration [25, 26, 30, 31, 34]. For example, in a recent community-based study of 266 RLS patients who had been treated with dopaminergic agents, only 25% had no evidence of augmentation [35]. Other common side effects include somnolence and general toxicity (all RLS medications); impulse control disorders (dopamine-receptor agonists); dyskinesias (dopaminergic agents), nausea and vomiting (dopaminergic agents, opioids); mood disturbances (α2δ ligands, opioids, benzodiazepines); weight gain (α2δ ligands); addiction (opioids, benzodiazepines); increased risk for falls (opioids, benzodiazepines), and other adverse sequelae [25, 26, 30, 31, 34, 36–44]. In addition, these effects can be particularly problematic in older adults [45, 46], who suffer disproportionately from RLS [42, 47]. In addition, the efficacy of all RLS medications commonly diminishes with time [31, 48–51], leaving patients with few treatment options. Given these drawbacks, investigation of safe, sustainable, nonpharmacologic therapies, that may not only alleviate RLS symptoms, but address associated comorbidities and apparent risk factors, is clearly warranted.

However, despite current clinical guidelines, including recent recommendations from the International RLS Study Group Task Force that medications be used only to treat “clinically significant symptoms that cannot be effectively managed behaviorally” [31], behavioral treatments remain largely untested and rarely implemented in practice. Specifically, although lifestyle changes and relaxation therapies, including yoga, are recommended for those suffering RLS [10], rigorous supporting research is lacking. To our knowledge, aside from our pilot studies [15, 52] only three small published trials have examined the potential benefits of lifestyle/behavioral interventions for individuals with RLS. These include a pre-post trial of cognitive behavioral therapy in 25 adults with primary RLS and psychosocial impairment, and two studies evaluating the effects of a 12–16-week exercise program vs. usual care on RLS symptoms: a non-randomized controlled trial in 14 hemodialysis patients [53] and a randomized controlled trial (RCT) of 23 community-dwelling older adults [54]. All reported significant improvement in RLS symptoms among participants assigned to the active intervention vs. the control group [53, 54] or baseline [55], suggesting that behavioral therapies may benefit those with RLS.

### Study rationale

Supported by our strong preliminary findings, drawing on the strengths of multiple disciplines, and testing a novel, clinical treatment paradigm, the proposed study will be the first RCT to rigorously assess the feasibility and acceptability of a promising mind-body therapy (yoga) for alleviating RLS symptoms and symptom burden. The study will provide essential preliminary data for a larger RCT to assess the efficacy of yoga for RLS management, and to evaluate the long-term effects of this novel therapy. Most important, this study will, in providing the foundation for the first rigorous trial of a novel behavioral therapy for RLS, have the potential to significantly alter the clinical management of this common and burdensome disorder.

As detailed above, medications used in RLS management carry risk of serious side effects that are especially problematic for older adults, and are often inappropriate for long-term use. Clearly, there is a need to investigate potentially safer, sustainable, low-cost therapies that are suitable for long-term use, and that have the potential not only to attenuate RLS symptoms, but to address the common comorbidities that may both result from, and contribute to, RLS. Particularly urgent is the investigation of promising patient-centered therapies that may enable and encourage patients to effectively manage their own health. While current guidelines indicate that behavioral management should comprise first-line treatment for RLS, and yoga and other relaxation therapies are sometimes recommended to RLS patients [56], rigorous supportive research is lacking. Findings from our two small, proof-of-concept studies suggest that yoga may significantly reduce RLS symptoms, improve sleep, and enhance mood, and decrease blood pressure in adults with RLS; observed effect sizes in these small trials were similar or superior to those reported in RLS drug trials [57–63], suggesting that yoga may offer a viable, multi-faceted, low-cost new treatment that is safer and more suitable for long-term use, and that has the potential to address symptoms as well as contributing factors.

An ancient mind-body discipline designed to promote physical, emotional, and mental well-being [64], yoga continues to gain popularity in the US, with over 13 million Americans reporting use of yoga in 2007 [65]. Our recent exploratory studies suggest that yoga may attenuate RLS symptoms, improve sleep, enhance mood, decrease stress, and reduce sympathetic activation in adults with RLS [15, 66], with observed effect sizes similar to, or greater than, those reported in trials of established drug treatments [57–63]. Numerous studies have shown yoga to improve quality of life, enhance well-being, and reduce pain, outcomes of clear relevance to RLS sufferers [67–75]. In addition, evidence from controlled trials by our group and others suggest that yoga may also improve indices of metabolic and autonomic function linked to both RLS and CVD risk [2, 9, 76–80]. Yoga has logistical advantages as well. Typically, a gentle practice with no appreciable side effects, yoga is relatively simple to learn and inexpensive to implement, and can be safely performed by a broad range of populations, including chronically ill, elderly, and even disabled adults [81–86]. The practice of yoga often brings immediate positive benefits, including feelings of relaxation and tranquility, helping to encourage continued adherence [77]. However, despite the promise of yoga as a safe, sustainable, cost-effective treatment for RLS, rigorous controlled studies are lacking.

### Design

This study will be a pilot, parallel-arm, community-based, RCT to assess the feasibility and acceptability of a 12-week yoga program vs. a 12-week educational film program for the management of RLS in adults aged ≥ 18 years with confirmed moderate to severe RLS. The protocol addresses the elements described in the Standard Protocol Items: Recommendations for Interventional Trials (SPIRIT) checklist (Additional file 1).

### Objectives

#### Primary objective

Our primary goal is to assess the acceptability and feasibility of a 12-week yoga vs. educational film program for RLS management in adults with moderate to severe RLS. Primary aims are as follows:

- Aim.1a. Assess participant recruitment and enrollment rates and determine optimal recruitment strategies. Reasons for refusal will be recorded and the information used to develop and implement strategies to address these barriers where appropriate
- Aim.1b. Determine participant retention rates and adherence in the yoga and educational film groups, with 80% retention, 80% class attendance, 75% home practice (yoga group), and 80% completion of weekly logs serving as benchmarks for acceptability. Information on barriers to adherence will be collected and used to inform ongoing strategies to enhance compliance

#### Secondary objectives

Our secondary objectives include the assessment of treatment fidelity in the two programs, as well as participant willingness to enroll in yoga programs of varying duration and intensity. In addition, we will measure RLS symptom severity, sleep quality, mood, and health-related quality of life at baseline and 12 weeks to obtain effect size estimates for these exploratory outcomes. This information will provide critical data on which to base our planned larger RCT to determine the efficacy of yoga as a potential therapy for RLS. Secondary aims are as follows:

- Aim.2.1. Monitor and assess treatment fidelity in the yoga and educational film programs, using specific metrics to gauge fidelity. Lapses in fidelity will be recorded and the data used to inform strategies to address these lapses
- Aim.2.2. Assess the effect sizes at week 12 of a beginner yoga and a structured educational film program for RLS symptoms and related key exploratory outcomes (sleep quality, mood, perceived stress, and health-related quality of life) in adults with moderate to severe RLS, using well-validated, self-report instruments commonly employed in evaluating RLS treatments. Effect size estimates for these exploratory outcomes will provide critical data on which to base sample sizes in future larger trials, as well as provide important preliminary information on sensitivity of specific endpoints to change with a 12-week yoga intervention
- Aim.2.3. Determine acceptable doses of yoga for a trial by assessing participant willingness to complete, as well as preference for yoga programs of the following duration and intensity: 8 weeks, 16 classes (2x/week); 12 weeks, 16 classes (2x/week for first 4 weeks, 1x/week thereafter); 16 weeks, 16 classes (1x/week). Acceptability and preferences will be evaluated using a self-report questionnaire administered to all participants at the follow-up assessment visit

## Methods

### Participants

**Fig. 1:**
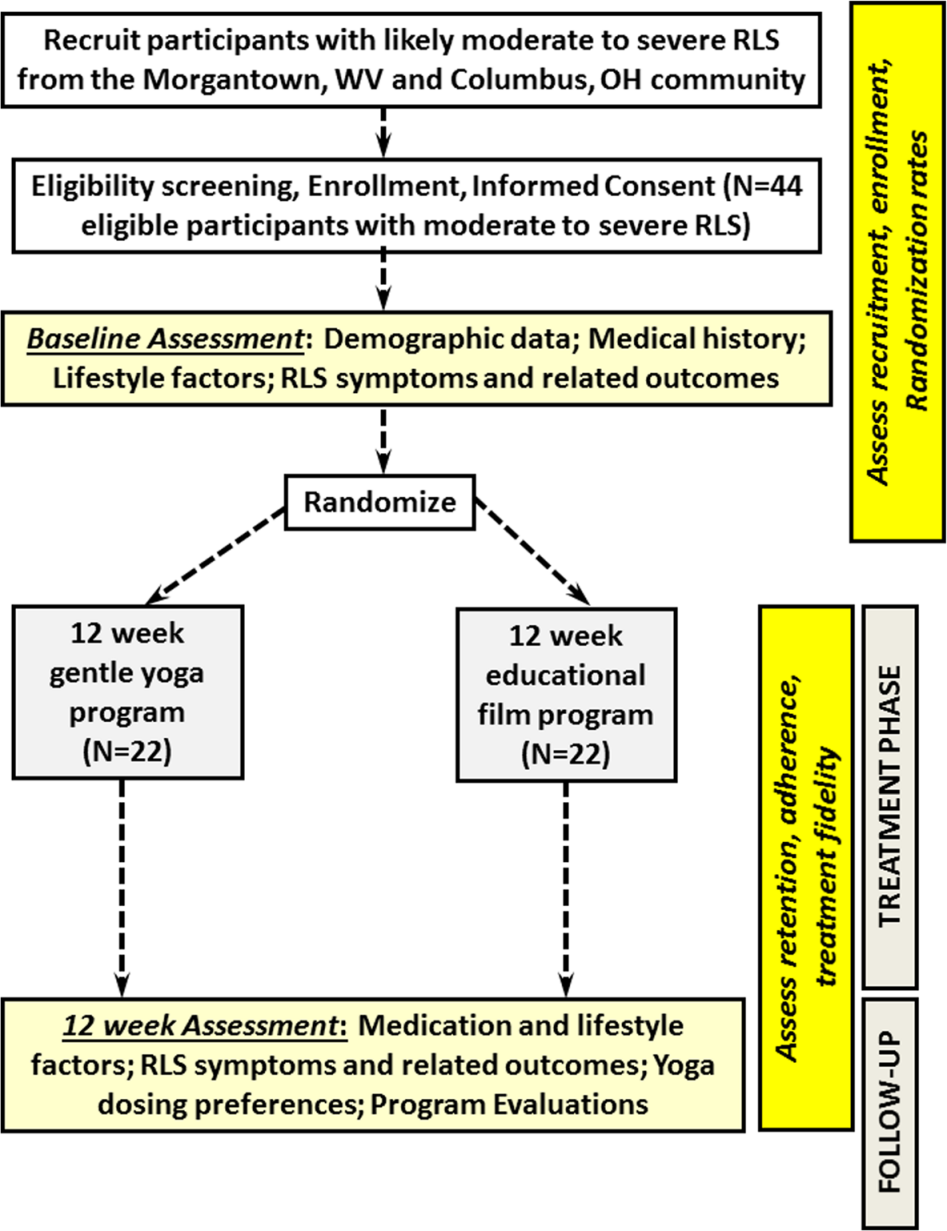
Study flow diagram

Forty-four ambulatory, overall healthy adults aged ≥ 18 years with confirmed moderate to severe RLS will be recruited and randomized to a 12-week beginner yoga program (*n* = 22) or a standardized 12-week educational film program (*N* = 22), modeled on interventions used in our previous trials [15, 87] (see Fig. 1).

#### Inclusion criteria

Study participants will be ambulatory adults ≥ 1 years of age with moderate-severe RLS, defined as:

1. Symptoms that meet all five of the International RLS Study Group’s essential diagnostic criteria for RLS [1, 88] (i.e., (1) an urge to move the legs, often associated with discomfort or disagreeable sensations in the legs, that (2) begins or worsen during periods of rest or inactivity; (3) is partially or totally relieved by movement; (4) is worse or only occurs in the evening or night; and (5) is not solely accounted for by another condition (e.g., leg cramps, positional discomfort, habitual foot-tapping))
2. RLS symptoms at least 1x/week for the previous 3 months
3. A score of at least 2 points (moderate) IRLS question 6: “How severe was your RLS as a whole?” [89, 90]; and
4. Confirmation of RLS diagnosis and study eligibility by a physician trained in sleep medicine; these criteria will help ensure exclusion of mimics (e.g., leg cramps, positional discomfort, arthritis pain, etc.) [20, 91]. Additional criteria include: willingness and ability to complete the 12-week yoga or educational film program and all study assessments; and willingness to avoid use of any new drugs or treatments other than the assigned intervention.

#### Exclusion criteria

Exclusion criteria are as follows: practiced yoga within the past year; currently on anti-psychotic medication; changed dosage of dopaminergic agents (e.g., levodopa, ropinirole, pramipexole) or any other central nervous system agents (e.g., sedative hypnotics, GABA analogs, narcotic analgesics, antiadrenergic agents, or anticonvulsants) within the past 3 months; hemoglobin (Hb) < 12 g/dL for women,< 13 g/dL for men; any orthopedic, neurologic, or other condition that might prevent safe completion of a 12-week yoga program or confound assessments (e.g., neuropathy; Parkinson’s disease; stroke; rheumatoid arthritis; renal failure; sleep apnea; recent (within the last 6 months) myocardial infarction; heart failure; cancer (other than non-melanoma skin cancer); pregnancy, or within 6 months post-partum). These medical exclusion criteria are consistent with those commonly employed in trials of RLS drugs and other RLS interventions [54, 92–96].

### Sample size

We performed a simulation study to determine the total sample size required to derive precise estimates of effect sizes for a two-arm randomized trial using a 1:1 randomization scheme for the primary intervention (yoga) and the educational film program; estimates were based on regression models using multiple simulations and scenarios, and specifically accounting for heterogeneity of effects. We considered seven combinations of effect sizes at 23 different sample sizes for total of 161 effect and sample size combinations. For each combination we ran 5000 simulations for a total of 805,500 simulations. The seven effect-size combinations were structured under the conservative assumption that all interventions would lead to a small to moderate improvement in outcomes and that the yoga intervention would lead to an improvement equal to or greater than the comparator condition. We used repeated simulations to determine the required post-attrition total sample size needed to obtain precise estimates of all three treatment effects under an intent-to-treat (ITT) analysis of variance (ANOVA) analysis. Based on findings of the simulation study and assuming 20% attrition, a sample size of 44 participants will permit reasonably precise estimates (within 95% confidence intervals of the true effect size value) for the two interventions, as well as reasonable estimates of recruitment, randomization, and adherence rates; moreover, estimates remain robust even at 30% attrition. Thus, the study will be adequately powered even if effect sizes for the yoga program are substantially smaller, and attrition rates greater than observed in our pilot studies [15, 66].

### Recruitment and study setting

Participants will be recruited from two sites: Morgantown, WV metropolitan area (WVU site) and Columbus, Ohio (The Ohio State University (OSU) site). In the WVU site, we will mail recruitment letters to all patients visiting a Morgantown area University Health Associates (UHA) clinic, who received a diagnosis of RLS (ICD-9 code: 333.94, 333.99) during the 5 years preceding study onset, as identified by electronic visit data. Consistent with Institutional Review Board (IRB) and Health Insurance Portability and Accountability Act (HIPAA) guidelines, the mailing list was obtained based on IRB approval of a waiver of research participants’ authorization for the use/disclosure of information. Only the minimum necessary information needed to identify potentially eligible subjects was accessed (i.e., name and mailing address). At both sites, we are recruiting from the community using local television, radio, and print media advertisements, as well as flyers and brochures posted in public places such as community bulletin boards (physical and electronic); health center waiting areas, and intranets. Study staff will also give presentations regarding the trial to interested lay and professional groups in the area, including clinician, support, university extension, and church groups, as well as other regional organizations and conferences. These strategies have proved effective in recruiting participants for previous studies of yoga-based interventions by WVU investigators in Morgantown, WV and by our partner investigators at OSU in Columbus, OH [52, 97–102].

Prospective study participants are being invited to contact the WVU study coordinator for further information. A member of the investigative team will describe the study, including eligibility criteria, to each potential participant over the telephone; those still interested will then be scheduled for a screening interview and baseline assessment, and sent a copy of the consent form to preview at their leisure prior to their first appointment.

### Consent and screening

We plan to enroll a minimum of 15 participants for each study cohort; within each cohort, participants will complete their baseline and follow-up assessments within the same 30-day window, and those in the yoga and film groups will begin and complete their respective classes at the same time. All class schedules will be fixed prior to randomization; as in our previous trials, every effort will be made to accommodate participant availability and preferences with respect to class timing.

At visit 1, an investigator will review the informed consent form with each participant in a private room; individuals still interested in participating in the study will sign the consent form. Each participant will sign two copies of the consent form, one to be given to the participant, and one to be stored with the study documents in a locked cabinet in a secure room in the WVU School of Public Health. Participant eligibility will be assessed using the IRLS (question 6) [89, 90] and a standardized screening checklist, similar to that used in our previous trials, which covers all of the eligibility criteria. Hemoglobin (Hb) levels will be assessed noninvasively, using the Masimo Pronto, a simple, hand-held pulse oximetry Hb monitor that has demonstrated excellent reliability and precision in both clinical and healthy populations [103–106]. Reasons for ineligibility and for non-participation of eligible candidates will be documented in a screening log.

Screening evaluations will occur within 30 days prior to the start of the intervention. Participant eligibility will be assessed using a standardized screening checklist similar to that used in our previous trials, which covers the inclusion and exclusion criteria (including RLS diagnostic criteria and at least moderate RLS severity); Hb levels will be assessed using the Masimo Pronto, a simple, hand-held pulse oximetry Hb monitor that has demonstrated excellent reliability and precision [103–106].

### Interventions

#### Yoga program (active intervention)

Participants randomized to the yoga group will complete a gentle *Iyengar* yoga program based on that developed and successfully implemented in our pilot studies [15, 66]. The yoga program was finalized following detailed review and input from an *Iyengar* Master Trainer.

Program logistics: yoga group participants will attend two 75-min classes per week for the first 4 weeks, then one 75-min class for the remaining 8 weeks, and will be asked to complete a 30-min homework routine 5 days/week on non-class days. Yoga classes will be held at two yoga studios in close proximity to the WVU Health Sciences Center campus (WVU site) and OSU Center for Integrative Health and Wellness (OSU site), respectively. As in our previous trials [15, 66], each yoga class will begin with simple yogic centering and breathing exercises, followed by a sequence of active and restorative poses, and ending with a 10-min guided supine relaxation practice. The yoga routines, including a collective total of 25 common *asanas*, have been tailored for sedentary adults naïve to yoga, gradually increasing in difficulty as students progress. Pose modifications and props (e.g., chairs, blankets, and straps) will be used as needed to enable participants to perform the poses safely and easily, regardless of their level of physical ability or fitness. In addition, each class will be restricted to no more than 12 participants to allow for personalized attention. The home practice (see Table 1) will be performed with the aid of a yoga DVD and a comprehensive, indexed training manual illustrating the homework routines; These guides are based on materials developed for our preliminary trials and modified to reflect the extension to a 12-week intervention period for the proposed study. Participants will also be provided yoga mats and straps to facilitate home practice.

**Table 1:** Yoga home practice routines

Home Practice 1: Weeks 1–3 (*use wall for side-angle standing poses*)	
1. Upward extended-leg pose	*Urdhva Prasarita Padasana*
2. Straight-leg pose: (a) bent knee 3x, (b) straight leg 2x	*Supta Padangusthasana*
3. Upward extended arms in mountain pose: (a) palms forward, (b) palms facing	*Urdhva Hastasana in Tadasana*
4. Extended side-angle pose	*Utthita Parsvakonasana*
5. Half-intense pose: (a) hands to wall, (b) hands on chair	*Ardha Uttanasana*
6. Extended triangle pose	*Utthita Trikonasana*
7. Extended one-foot frog pose	*Utthita Eka Pada Bhekasana*
8. Seated wide-angle pose	*Upavistha Konasana*
9. Bound-angle pose	*Baddha Konasana*
10. Seated twist pose (in chair)	*Bharadvajasana*
11. Corpse pose	*Savasana*
12. Corpse pose with breathing	*Savasana Pranayama*
Home Practice 2: Weeks 4–6 (*use wall for side angle standing poses*)	
1. Upward extended-leg pose	*Urdhva Prasarita Padasana*
2. Straight-leg pose: straight leg 3x	*Supta Padangusthasana*
3. Upward extended arms in mountain pose: palms facing coming up from the side	*Urdhva Hastasana in Tadasana*
4. Extended side-angle pose	*Utthita Parsvakonasana*
5. Half-intense pose: hands to the wall	*Ardha Uttanasana*
6. Warrior II pose	*Virabhadrasana II*
7. Extended triangle pose	*Utthita Trikonasana*
8. Intense pose: buttocks on the wall and hands on books	*Uttanasana*
9. Extended one-foot frog pose	*Utthita Eka Pada Bhekasana*
10. Thunderbolt pose	*Vajrasana*
11. Seated wide-angle pose	*Upavistha Konasana*
12. Seated twist pose (in chair) (2 blankets)	*Bharadvajasana*
13. Supported bridge pose (2 blankets)	*Setubandha Sarvangasana*
14. Corpse pose: feet to the wall	*Savasana*
15. Corpse pose with breathing	*Savasana Pranayama*
Home Practice 3: Weeks 7–9 *(no wall for side angle standing poses)*	
1. Upward extended-leg pose	*Urdhva Prasarita Padasana*
2. Straight-leg pose: straight leg	*Supta Padangusthasana*
3. Fierce pose: back to wall	*Utkatasana*
4. Extended side-angle pose	*Utthita Parsvakonasana*
5. Half-intense pose: hands on books	*Ardha Uttanasana*
6. Warrior II pose	*Virabhadrasana II*
7. Extended triangle pose	*Utthita Trikonasana*
8. Intense pose: holding elbows	*Uttanasana*
9. Downward-facing dog pose: (a) hands at wall, (b) heels on wall	*Adho Mukha Svanasana*
10. Extended one-foot frog pose	*Utthita Eka Pada Bhekasana*
11. Thunderbolt pose	*Vajrasana*
12. Staff pose	*Dandasana*
13. Seated wide-angle pose	*Upavistha Konasana*
14. Bound angle pose (2 blankets)	*Baddha Konasana*
15. Seated twist pose (in chair)	*Bharadvajasana* (on blankets)
16. Supported bridge pose	*Setubandha Sarvangasana*
17. Corpse pose	*Savasana*
18. Corpse pose with breathing	*Savasana Pranayama*
Home Practice 4: Weeks 10–12 *(no wall for side angle standing poses)*	
1. Upward extended-leg pose	*Urdhva Prasarita Padasana*
2. Straight-leg pose: straight leg 1x	*Supta Padangusthasana*
3. Fierce pose: feet apart	*Utkatasana*
4. Extended side-angle pose	*Utthita Parsvakonasana*
5. Intense pose: holding elbows	*Uttanasana*
6. Downward-facing dog pose	*Adho Mukha Svanasana*
7. Warrior I pose: back heel at wall	*Virabhadrasana I*
8. Hero’s pose	*Virasana*
9. Garland pose: (a) hold elbows, (b) extend arms	*Malasana*
10. Bound angle pose	*Baddha Konasana*
11. Head-to-knee pose	*Janu Sirsasana*
12. Western intense pose	*Paschimottanasana*
13. Active bridge pose	*Chatush padasana*
14. Corpse pose	*Savasana*
15. Corpse pose with breathing	*Savasana Pranayama*

#### Educational film program (comparator)

Participants assigned to this group will attend one 75 min class per week for 12 weeks, and will be asked to complete a daily log at home recording any RLS or sleep treatments they tried based on what they learned in class or for any other reason. At the first class, film group participants will receive a comprehensive set of lay educational materials regarding RLS symptoms, causes, and epidemiology and detailing nonpharmacologic strategies for RLS management based on current national guidelines, including recommendations regarding sleep hygiene, lifestyle modifications, and behavioral approaches. Each class will be held at the WVU Health Sciences Center, and include a brief meet-and-greet period at the start of class, an instructional film segment of approximately 60 min, and a 10–15-min group discussion, facilitated by a health educator familiar with sleep disorders and common nonpharmacologic treatments. While sitting for 75 min/week is unlikely to lead to worsening of symptoms in this largely sedentary population, we will, as in our previous trials, encourage participants to stand and/or stretch during the film and discussion if they so desire. All participant comments will be noted, and, together with information provided on the exit questionnaire, will be used to refine the program for the larger trial. Upon study completion, film group participants will receive all yoga homework materials, and be offered a half-day workshop in yoga for RLS.

Course content for this standardized film education program will include information on: RLS symptoms, epidemiology, and management, including sleep hygiene practices; other sleep disorders and associated comorbidities, and on mind-body and other complementary therapies likely to be of interest to those taking part in a yoga and sleep education study. As in our previous RCT [87], educational films comprise the mainstay of the instructional program, allowing participants to be informed by nationally-recognized subject experts on a variety of topics in a professional and entertaining way. This 12-week program is designed to be easily replicable and to ensure reasonable comparability of staff attention and social interaction. The film selection procedure was similar to that successfully employed in our earlier studies; 41 commercially available documentary and educational films were screened for content relevance, accuracy, and likely appeal to the target study population. Each film was reviewed by three or more members of the study team, and films for inclusion in the program were selected by consensus. The program includes 11 films, including three regarding sleep, and one specific to RLS (see Table 2).

**Table 2:** Educational film program

	Special section or timing cues
*Session 1*	
Introduction to the study and distribution of restless legs syndrome (RLS) information packets	
Restless Leg Syndrome: An Uncontrollable Urge to Move (2012) (Part of the award-winning series: Healthy Body, Healthy Mind)	
Good Night with the Sleep Doctor DVD (2008) (Dr. Michael Breus)	Act 1 [end at 25:15]
*Session 2*	
Good Night with the Sleep Doctor DVD (2008) (Dr. Michael Breus)	Acts 2 and 3
*Session 3*	
10 Things You Should Know About Sleep (2009) (A BBC production)	
*Session 4*	
The New Medicine (2006) (FREDDIE award winner for excellence in health and wellness media)	Part 1 [3:37–56:30]
*Session 5*	
Mindfulness and Meditation: Stress Reduction (2000) (Jon Kabat-Zinn)	
*Session 6*	
Stress and Relaxation Explained: An Introduction to Stress Management and Relaxation Techniques (2007)	Introduction; Part 1 (Stress): Explanations: Nature of Stress, Biological Origins, Symptoms, Health Effects; Part 2 (Relaxation): Relaxation Techniques: Mind-Body Medicine, Managing \Stress, Relaxation Techniques; Real World Balance; Part 4 (Guided relaxation)
*Session 7*	
Yoga Unveiled: The Evolution and Essence of a Spiritual Tradition (2004)	Part 5: Yoga as Therapy
*Session 8*	
UCSF’s Mini Medical School for the Public – Health and Vitality: What Science Tells Us About How to Thrive (2011)	
*Session 9*	
The Connection (2014)	Main section
*Session 10*	
Happiness 101 with Tal Ben-Shahar (2009)	Main section
*Session 11*	
The Real Age Makeover with Michael F. Roizen, MD (2005)	Part I
*Session 12*	
The Real Age Makeover with Michael F. Roizen, MD	Q&A [to 24:08]
Happiness 101	Sloat bonus material
The Connection	Benson Interview

#### Criteria for discontinuing or modifying allocated interventions for a given trial participant

Each class will be restricted to no more than 12 participants to allow for personalized attention. In the event that a participant experiences an adverse event (AE) which makes continuation of the intervention unsafe for that individual, the study coordinator will inform the principal investigator (PI) and the study physician, referencing just the participant’s study ID number, and the intervention will be discontinued for that participant. With their permission, participants will continue to be followed if the study intervention is discontinued. Any AE requiring discontinuation that is judged to be due to the intervention will prompt re-evaluation and possible modification of the intervention.

#### Strategies to improve adherence to intervention protocols and procedures for monitoring adherence

Attendance data and homework logs will be collected each week by the group instructor, and monitored for non-adherence. Instructors will query non-adherent participants to ascertain reasons for non-adherence and determine appropriate strategies to approve adherence (e.g., scheduling a different time of day for performing or recording homework, using yoga props or modified versions of poses, following the yoga routine from previous weeks if the current routine is too challenging or problematic, etc.).

#### Relevant concomitant care and interventions that are permitted or prohibited during the trial

As noted above, exclusion criteria include the use of anti-psychotic agents and change in dopaminergic or other central nervous system agents within the previous 3 months; any change in medication use or dosage during the trial will be monitored. In addition, participants are asked to avoid the use of any new drugs or treatments other than the assigned intervention, and are not allowed to participate in another intervention study for the duration of the trial. No concomitant interventions are required.

### Instructors

All study yoga instructors have been certified in *Iyengar* yoga (≥ 500 h), with at least 3 years of experience teaching adults with a range of chronic health conditions and trained in the final RLS yoga protocol by lead study yoga teacher, Dr. Kimberly Williams. Instruction will be aided by an illustrated training manual and reference guide based on our pilot work and provided to each instructor along with the home-practice DVD.

The film intervention will be administered by a health educator versant in sleep disorders and an advanced graduate student trained for this purpose. This program, based predominantly on educational films, is designed to be easily replicable. The instructor’s role is primarily to facilitate group discussion of the content presented in the films.

### Assignment of interventions

#### Allocation and concealment

The statistician, who will have no contact with the participants, generated a randomized assignment master list and provided sequentially numbered opaque envelopes containing the group assignment and corresponding study forms. The consenting team member will assign the unique number from the next envelope in sequence as the participant’s study ID, and enter the number and participant name on a secure electronic database; this information will be linked to the randomization assignment, with the linked data stored in a separate password-protected database. The sealed, numbered assignment packet will be given to the participant at the conclusion of visit 1 after collection of baseline data.

#### Randomization

Randomization will occur immediately following completion of baseline evaluations and no more than 30 days prior to initiation of the intervention. Eligible participants will be randomized using a 1:1 ratio, to the yoga (*N* = 22) or educational film group (*N* = 22), based on an allocation sequence generated by the study statistician using a randomly varying block randomization method to ensure equal distribution among treatment groups [107].

#### Blinding

The majority of study personnel will be blinded to treatment assignment until the database is deidentified and locked. The randomization scheme will be devised and provided by an individual with statistical expertise who has no access to the data or influence on study outcomes; this individual will maintain the master coding sheet indicating the treatment assignment corresponding to each participant number. Following confirmation of eligibility and baseline assessment, randomization will be performed by the consenting team member, who will have no advance knowledge of treatment allocation; sealed, opaque envelopes containing information regarding the participant’s assigned intervention will simply be uniquely numbered, with no external indication of treatment assignment; as in previous studies, participants will be instructed to open their envelopes upon exiting the building. The study coordinator will maintain the list linking participant numbers with personally identifiable information. Assessments will likewise be conducted by trained study personnel blinded to treatment allocation and specifically hired for this purpose. The yoga and educational film group instructors will be responsible for documenting attendance and collecting homework logs, as well as for contacting participants. Instructors will not have access to the data or be involved in data monitoring or analysis. Data will be entered (double-keyed) into an existing data template by two students trained in data management; participants will be identified only using a unique number.

In the unlikely event that there are three serious adverse events (SAEs) in either group that are judged to be related to the intervention, the study coordinator will inform the PI of this fact. Up to this point, the PI will be aware of all SAEs but will be unaware of the affected participant’s group assignment. If the PI agrees that the SAEs are related to the intervention, she will determine that the blind will be broken. In that event, the study coordinator will give the list linking participant numbers with personally identifiable information to the PI.

### Data collection and outcomes

Assessments will be conducted by study team members with training and experience in clinical assessment; all assessment personnel will be blinded to treatment assignment. Baseline data, including that on exploratory outcomes, will be collected prior to randomization (see “Patient timeline” below).

To determine feasibility and acceptability of the 12-week yoga and educational film comparator program, our primary aim, we will collect comprehensive information, both overall and by treatment group, on: rates of recruitment and success of different recruitment strategies, and reasons for refusal to participate; screening, randomization and enrollment rates, including reasons for screening failures and refusals to enroll/be randomized to treatment; rates of retention (completion of assessment visits) and adherence (completion of home logs and class attendance rates (yoga and film groups); completion of home practice (yoga group)); and participant satisfaction, assessed via a structured *program evaluation questionnaire* regarding participant perceptions of, and experiences with, the study and their respective programs. Attendance data will be collected by the instructor for each group, as will homework logs. Participants will complete the program evaluation questionnaire, modeled on that used in our previous trials [15, 87, 108], upon completion of the 12-week intervention period or upon leaving the study.

A structured questionnaire regarding possible yoga-dosing scenarios will be administered at week 12 to determine participant preferences relative to yoga programs of varying duration and intensity (8 weeks, 16 classes (2x/week); 12 weeks, 16 classes (2x/week for the first 4 weeks, 1x/week thereafter); 16 weeks, 16 classes (1x/week), as well as time of year that the classes meet. Information from this questionnaire will address a secondary aim, and aid in optimizing the yoga protocol for our future studies.

Data on exploratory outcomes and certain modifying/confounding factors will be collected at baseline and 12 weeks to determine the effect sizes of the programs, data critical to the design of the planned larger RCT. RLS symptom severity, the primary efficacy outcome for the full-scale trial, will be evaluated using the International RLS Rating Scale (IRLS), a 10-item scale which includes questions related to frequency, intensity, and impact [89]. Considered the gold standard for measuring RLS symptoms [109, 110], this instrument is recommended for use in RLS clinical trials by the European RLS Study Group (EURLSSG) [111], and is widely used both in the US and internationally [3, 112]. Additional exploratory outcomes are sleep quality, mood, and health-related quality of life (HrQOL), endpoints recommended for inclusion in all clinical trials of RLS [26, 111]. These outcomes will be assessed via a short battery of self-report instruments commonly used in pharmaceutical trials of RLS patients [113–116]. Sleep quality will be assessed using the nine-item Pittsburgh Sleep Quality Index (PSQI) [117]; mood will be evaluated using the 65-item Profile of Mood States (POMS) [118] and 10-item Perceived Stress Scale (PSS) [119, 120]; and HrQOL will be measured using the 36-item MOS Short Form-36 (SF-36) [121, 122]. These self-report instruments are well-established scales that have been shown to be sensitive to short-term behavioral interventions, and have been validated in a wide range of populations, including RLS patients [118, 123–137]. As in our previous trials [15, 66] we will also assess potential changes in blood pressure and heart rate to determine effect sizes in these indices of sympathetic activation; heart rate and blood pressure will be measured three times at each assessment using an automated blood pressure monitor (Omron HEM-780) after a 5-min seated rest period.

To measure change in social support and physical activity, potential modifying or confounding variables, participants will complete the 11-item Duke Social Support Index (abbreviated form [138, 139]) and 10-item Physical Activity Scale for the Elderly [140–142] (to capture physical activity other than yoga) at each visit; information on Body Mass Index (BMI, calculated as weight(kg)/height(m)^2^), caffeine and alcohol consumption, smoking, and use of non-RLS medications will also be collected at each assessment. To assess *expectation of benefit,* the Credibility/Expectancy Questionnaire (CEQ) [143, 144] will be collected from participants after their first class. Participants will be asked to record use of other RLS treatments on their weekly logs.

#### Patient timeline (Fig. 2)

**Fig. 2:**
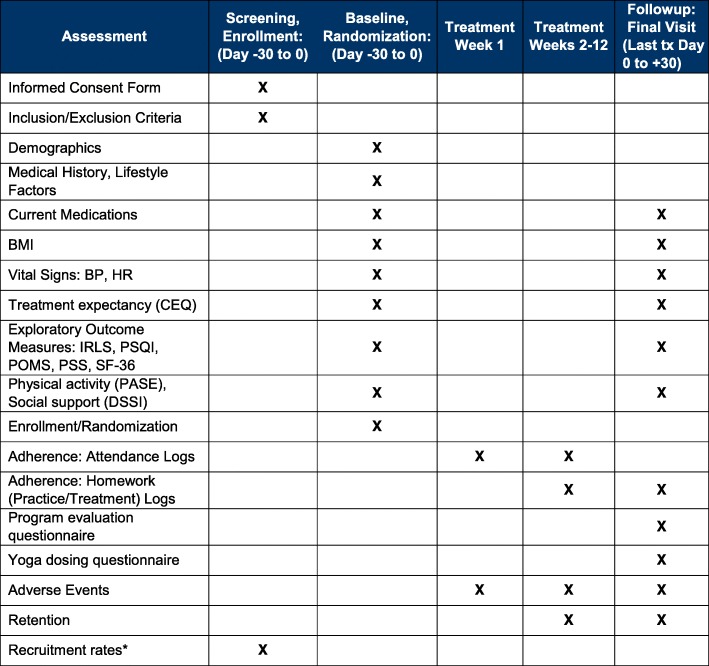
Schedule of evaluations. *To be monitored continuously throughout study until study is closed to enrollment. Abbreviations: *BP* blood pressure, *BM*I Body Mass Index (weight(kg)/height(m)^2^), *CEQ* Credibility/Expectancy Questionnaire, *HR* heart rate, *DSSI* Duke Social Support Index, *IRLS* International Restless Legs Scale, *PASE* Physical Activity Scale for the Elderly, *POMS* Profile of Mood States, *PSQI* Pittsburgh Sleep Quality Index, *PSS* Perceived Stress Scale, *SF-36* 36-item MOS Short Form-36, *Tx* treatment

### Data management

Completed data forms or other hard-copy documents containing protected health information will be kept in a locked file in an investigator’s office. Data will be entered into an electronic deidentified database by authorized study team members, checked for completeness and accuracy. The PI or study staff will review all data collection forms on an ongoing basis for data completeness and accuracy as well as protocol compliance. Access to data with identifiers will be restricted to authorized study team members and regulatory authorities. Data verification will be performed by someone other than the individual originally collecting the data, or by double-data entry.

Electronic data will be stored on a secure server which is maintained, with regular back-ups, by WVU Health Sciences Center system administrators and accessible from password-protected computers in the WVU Department of Epidemiology. Any data, forms, and other records with identifiers that leave the site will be transported in a locked file box to maintain confidentiality. Identifiable data will be destroyed 5 years after study completion or 3 years after the last publication based on when the data is published, whichever comes last (unless future regulations dictate that the data be kept for longer).

### Statistical methods

All analyses will be performed using SAS [145] or R [146]. For the primary feasibility aims, we will be evaluating recruitment and enrollment rates (all interested potential participants), acceptability of randomization (all enrolled subjects), retention, and adherence (all randomized participants). For the secondary aims, we will define the intent-to-treat (ITT) population as all subjects who are randomized with baseline assessments and at least one post-randomization assessment. The per-protocol analysis of the exploratory outcomes (RLS symptoms, sleep quality, mood, stress, and quality of life) will include all participants who completed both assessments and at least 75% of the classes.

*Feasibility of the proposed design* (*A.1*) will be evaluated in several ways:

*Recruitment*: we will examine recruitment time and success rates for different recruitment strategies and assess the percentage of interested volunteers who meet the study criteria, agree to participate and are randomized. We will use Fisher’s exact test to determine whether there are any differences in recruitment rates across the different strategies, and exponential regression model to assess the recruitment strategy on recruitment time.

*Retention*: will initially be assessed using descriptive statistics, including comparisons between retained individuals and drop-outs. The nonparametric Kaplan-Meier curve will be used to estimate the probability of retention over the duration of the study. If retention is unexpectedly low, we will use the proportional hazard model to assess retention differentials by treatment, demographic, and health-related factors to better target and retain specific groups in future trials.

*Randomization success*: (i.e., the two groups are similar demographically and on anthropometric and exploratory outcome measures at baseline) will be assessed using the one-sample run test. To evaluate *adherence* to the study protocol, we will determine missing-data rates at baseline and follow-up, class attendance (both groups), and completion of weekly logs.

*Treatment fidelity* (*A.2.1*) will be monitored and lapses recorded and compared between groups, along with outcomes of remediation strategies. We will use the Fisher’s exact (continuous data) and chi-square tests (using benchmark cut-offs) to determine potential differences in treatment adherence and fidelity.

*Treatment effect sizes* (*A.2.2*), based on between-group differences in change over time, will be estimated using one-way ANOVA models. Effect size estimates will be calculated using both ITT (primary) and per-protocol analyses. These unbiased estimates of intervention effect size will inform sample size determination for our planned future efficacy trial [147]. Scores on *the Yoga-dosing Questionnaire* (*A.2.3*) will also be assessed using descriptive statistics to determine optimal scheduling and maximum acceptable dosing of yoga for our future trial.

*Missing data*: every effort will be made to minimize missing data relevant to all aims. If missing data does occur and the missing-data pattern (probability model) is not missing completely at random (MCAR), then we will consider approaches appropriate for missing at random (MAR) and missing not at random (MNAR). If the pattern or structure of the plausible missing data suggests MAR, either the EM algorithm [148], inverse weighting [149], or multiple imputation (MI) [150, 151] will be used and the missing-data mechanism will be constructed from the collected data [150, 152, 153]. The selected method will reflect the appropriateness of the method to best estimate the underlying missing-data mechanism [148–150, 152]. Participant characteristics will be assessed, including demographic and lifestyle characteristics, RLS severity, medical history, health-related factors, and baseline questionnaire scores for the estimation of plausible models (i.e., MCAR, MAR, and MNAR) for the missing-data mechanism. While we will not be able to definitively test if the missing data are MNAR, If there are indicators suggestive of MNAR (e.g., drop-outs differ significantly from non-drop-outs in key characteristics), we will use MI in conjunction with a sensitivity analysis to provide a set of plausible conclusions recognizing that each conclusion will be predicated on the assumptions necessary for constructing a MNAR missing-data mechanism model [150, 153].

### Data monitoring, harms, and auditing

The frequency of data review for this study differs according to the type of data and can be summarized as follows: data on accrual, status of all enrolled subjects, adherence, and AEs will be reviewed by the PI and study coordinator every 4 months; accrual and AEs will also be reviewed yearly by the PI, IRB, Independent Monitoring Committee (IMC) (comprising three experts with appropriate expertise and experience in clinical trials, biostatistics, and clinical medicine), and NCCIH; SAEs will be reviewed per occurrence by all.

While both programs are minimal risk interventions, we are further ensuring participant safety by: using highly trained *Iyengar* instructors experienced in teaching adults with chronic health conditions and in adapting poses to minimize discomfort and risk of injury; including only gentle postures in the program; and maintaining small class sizes to ensure adequate personalized attention. In addition, all participants will be encouraged to report any AEs to study staff and on their yoga and co-intervention logs. Yoga group participants will also be queried about AEs by the yoga instructors during class. Yoga instructors will notify the study coordinator regarding any AE.

The study coordinator will promptly report AEs to the IRB according to protocol and IRB policies; complete an adverse events form and grade the AE; and contact the participant by telephone on a weekly basis to monitor the event until the event resolves, the study is completed, or the participant is removed from the study. In addition, subjects can choose to stop the study at any time. Any serious and unexpected AEs will require re-evaluation of the risk of the study. Monitoring and aggregate review will be performed by the PI, IRB, and IMC through annual review.

#### Stopping rules

This study will be stopped prior to its completion if: (1) the intervention is associated with adverse effects that call into question the safety of the intervention; (2) any new information becomes available during the trial that necessitates stopping the trial; or (3) other situations occur that might warrant stopping the trial. Because this RCT is specifically designed to be a feasibility study, and we will be investigating recruitment rates and optimal recruitment strategies as part of our primary aim, slow accrual would not comprise a justification for stopping the trial.

In addition, the study may be discontinued at any time by the IRB, NCCIH, the OHRP, or other government agencies as part of their duties to ensure that research participants are protected.

## Discussion

In summary, RLS is a common and burdensome sleep disorder associated with profound impairment of health, well-being, and quality of life, and with significant personal, societal, and economic burden [6, 7, 26]. There is no cure for RLS, and current front-line drug therapies can carry severe side effects. If, as our promising preliminary data suggests, yoga ultimately proves feasible and effective in reducing RLS symptoms and symptom burden, yoga will offer a novel, safe, and likely cost-effective approach to RLS prevention and management, targeting not only RLS symptoms, but also likely contributing factors and common comorbidities.

This study will lay the essential groundwork for a planned larger RCT to determine the efficacy of a yoga program for reducing symptoms and the associated burden of RLS, a common and potentially debilitating sleep disorder. Specifically, central goals of this trial include evaluating the feasibility and acceptability of a 12-week RCT of yoga vs. an attention control, and optimizing the yoga protocol for a planned larger trial of yoga for RLS management; we will also obtain effect size estimates for specific outcomes of relevance to RLS management, information critical to designing and powering our planned future study. Based on our strong pilot data, the study is consistent with the urgent national need to curtail spiraling health care costs and to identify sustainable, potentially cost-effective new approaches to chronic disease management. If the findings of the current trial and the subsequent larger RCT are positive, this study will also help support a new approach to clinical treatment of this challenging disorder, help foster improved understanding of RLS etiology, and ultimately contribute to reducing the individual, societal, and economic burden associated with this condition.

### Ethics and dissemination

This study has been approved by the West Virginia University Institutional Review Board (IRB#1505699758). No significant changes will be made to this protocol without the prior approval of the WVU IRB and the NCCIH. As described above, each participant will provide written informed consent prior to the initiation of baseline assessments or the intervention.

#### Confidentiality

During this study, medical history, vital signs, and assessment questionnaires will be completed at baseline and at regular intervals during the study. All of the material collected are for research purposes only, and data will be kept in strict confidence. Information will not be released without written permission of the participant, except as necessary for monitoring by the IRB, the NCCIH, the OHRP, and the IMC. Confidentiality will be ensured by use of identification codes. This data, collected at the assessment visits, will be identified with a study identification code unique to the subject.

#### Dissemination plans

Upon completion of the trial, the authors plan to present the study findings at conferences aimed at physicians typically treating patients with RLS, and at other integrative health care practitioners such as yoga therapists. In addition, the investigators plan to submit our findings for publication in relevant peer-reviewed journals; we do not intend to hire professional writers.

## Trial status

This trial is currently open for enrollment.

## Additional file

Additional file 1: Standard Protocol Items: Recommendations for Interventional Trials (SPIRIT) Checklist. (DOC 123 kb)

## Abbreviations

BMI: Body Mass Index (weight(kg)/height(m)^2^)
BP: Blood pressure
CEQ: Credibility/Expectancy Questionnaire
DSSI: Duke Social Support Index
HR: Heart rate
IRLS: International Restless Legs Scale
PASE: Physical Activity Scale for the Elderly
POMS: Profile of Mood States
PSQI: Pittsburgh Sleep Quality Index
PSS: Perceived Stress Scale
SF-36: 36-item MOS Short Form-36
Tx: Treatment
